# Psychopathological sequelae of female genital mutilation and their neuroendocrinological associations

**DOI:** 10.1186/s12888-018-1757-0

**Published:** 2018-06-13

**Authors:** Anke Köbach, Martina Ruf-Leuschner, Thomas Elbert

**Affiliations:** 1 Department of Clinical and Neuropsychology, University Konstanz, Universitätsstraße 10, 78457 Konstanz, Germany; 2 Vivo international e.V., Postfach 5108, 78430 Konstanz, Germany

**Keywords:** Female genital mutilation, FGM, Female genital cutting, Posttraumatic stress disorder (PTSD), Dissociation, Depression, Hair cortisol

## Abstract

**Background:**

Anecdotal evidence suggests the frequently traumatic nature of female genital mutilation (FGM). At present, systematic research on the psychological sequelae of this tradition has remained limited. The study provides preliminary, high-quality psychodiagnostic data on potential psychopathological consequences of FGM, with a focus on posttraumatic stress disorder (PTSD), shutdown dissociation and other stress-related variables.

**Methods:**

We investigated a convenience sample of *N* = 167 women, supported by the women’s affairs headquarters in Jijiga (capital of the Ethiopian Somali Region) and a local Ethiopian non-governmental organization. Our main outcome measures were PTSD (PSS-I) and shutdown dissociation (ShuD). We also assessed depression and anxiety (HSCL-25), major depression, substance abuse and dependence, suicidality and psychotic disorders (M.I.N.I.; sub-scales A., B., K., and L.). In addition, we collected hair samples to assess hair cortisol concentrations (HCC) as a neuroendocrinological measure.

**Results:**

The majority of women endured FGM (FGM I: 36%, FGM II/III: 52%) and, regardless of the level of the physical invasiveness, almost all women reported having felt intense fear and/or helplessness. FGM II/III, the more invasive form, was associated with a greater vulnerability to PTSD symptoms (*p* < .001) and shutdown dissociation (*p* < .001). Symptoms of depression (*p* < .05) and anxiety (*p* < .01) were also elevated. Random forest regression with conditional inference trees revealed evidence of an alteration of the cortisol levels in relation to the age when FGM was experienced (< 1 year) and the invasiveness of the procedure.

**Conclusion:**

More extensive forms of FGM are associated with more severe psychopathological symptoms – particularly with an increased vulnerability to PTSD. Higher hair cortisol levels in women who experienced FGM before their first year of age or had more severe forms of FGM indicate long-term neuroendocrinological consequences of FGM and trauma in general on the stress system.

## Background

Female genital mutilation (FGM) refers to all procedures that intentionally alter or harm the female genital organs for non-medical reasons [1]. The World Health Organization (WHO) has classified four major types: (1) *Clitoridectomy* (in the following referred to as FGM I) *is the partial or total removal of the clitoris and, in very rare cases, only the prepuce.* (2) *Excision* (in the following referred to as FGM II) *is the partial or total removal of the clitoris and the labia minora, with or without excision of the labia majora,* while (3) *infibulation* (in the following referred to as FGM III) *is the narrowing of the vaginal opening through the creation of a covering seal. The seal is formed by cutting and repositioning the inner, or outer, labia, with or without removal of the clitoris.* Defibulation—which usually takes place before sexual intercourse or childbirth—is the opening of the formerly infibulated part. In cultures that traditionally practice infibulation, women enter into a cycle of defibulations and re-infibulations (e.g., natural adhesion of the injured labia, infibulation after birth). (4) *Other practices include all other harmful procedures to the female genitalia for non-medical purposes,* e.g. *pricking, piercing, incising, scraping and cauterizing the genital area* [1]. International institutions such as the United Nations (UN) or WHO follow unambiguous anti-FGM policies. Still, more than 200 million women from countries in Africa and the Middle East are affected [2]. Adverse obstetric outcomes increase alongside more extensive FGM [3].

Anecdotal evidence from women who have endured FGM provides insight into the traumatic nature of the tradition [4], and recent studies have confirmed that the majority of women with FGM reported having felt intense fear, helplessness and pain during the procedure [5]. Besides the subjective validation of FGM as traumatic event, within the DSM-5 classification system, FGM applies to the A-criterion (threatened death, serious injury and/or sexual violence) of posttraumatic stress disorder (PTSD) [6]. PTSD constitutes a psychiatric condition that typically develops in response to (repeated) exposure to serious threats [6], whereby cumulative exposure is a reliable predictor of the intensity of PTSD symptoms (also indicated as building block effect; e.g., [7, 8]). PTSD patients typically suffer from the cognitive, emotional, and physiological re-experiencing of their traumatic event(s) (B-criterion), avoidance of trauma reminders (C-criterion), an alteration of mood and cognition (D-criterion), and hyperarousal (E-criterion) [6]. Comorbidities with depression, substance dependency, dissociation and other psychological disorders are frequent [9]. To date, only a few scientific studies have investigated the relationship between FGM and PTSD. In fact, Mulongo et al. [10] reviewed more than 1 thousand articles on FGM, of which only ten reported psychological outcomes and only two original studies met high scientific standards. Behrendt and Moritz [5] compared 23 Senegalese women with FGM to 24 in the noFGM group. Compared to the noFGM group, the FGM group presented a higher prevalence of PTSD (30% vs. 0%) as well as an increased prevalence of other anxiety and affective disorders. Moreover, Knipscheer et al. [11] recently investigated a sample of 66 immigrant women in the Netherlands. More than one third of this group presented with clinically relevant symptoms of PTSD, anxiety and/or depression.

Psychoneuroendocrinological research reveals the important role of the Hypothalamus–Pituitary–Adrenal (HPA) axis in the regulation of stress reactions in trauma-exposed individuals with and without PTSD [12, 13]. Consequently, serious stressors, such as FGM and its associated lifelong sequelae of physical [3] and psychological pain [5, 9] may permanently alter the function of the HPA axis [14]. Especially early life stress has been linked with epigenetic (re)programming of the HPA axis (e.g. [15]). Hair cortisol concentration (HCC), measured in hair that has grown over the last month or more, quantifies long-term cortisol secretion patterns. With it, HCC serves as physiological marker for stress-related changes [13, 16, 17]. Specific associations of the HCC with FGM have not been investigated previously. Relevant earlier studies found higher HCC in patients suffering from chronic pain [18], who experienced a greater number of traumatic events [19–21], and for those reporting more major life stressors [22]. In contrast, Steudte-Schmiedgen et al. [23, 24] reported a negative association with the number of lifetime traumatic events, and Hinkelman et al. [25] found lower HCC in patients with depression than in healthy controls (both of whom were exposed to traumatic experiences during childhood). In a recent review, Steudte-Schmiedgen et al. [13] suggests a two-stage model hypothesizing an initial increase in cortisol after the traumatic event followed by a gradual attenuation as the event becomes more distant. With it, the authors emphasize the importance of the temporal aspect of trauma exposure and HPA axis regulation together with the current level of stress.

We conducted psychological interviews with women who live in Jijiga, the capital of the Ethiopian Somali Region. In this region, FGM is still widespread, despite the endorsement of legal provisions on behalf of the Ethiopian parliament to protect girls subject to the tradition. The *Ethiopian Demographic and Health Survey 2005* (EDHS 2005) [26] reported that 97.3% of women who live in the Somali region had endured FGM. Regarding the traumatic nature of the practice, we hypothesized FGM to be associated with a heightened risk of PTSD and trauma-related shutdown dissociation. We also take into account depression, anxiety, substance abuse and dependence, suicidality and psychosis. Focusing on PTSD severity, we expected FGM to exacerbate the cumulative effect of the number of potentially traumatic event types [8]. Furthermore, we aimed to explore the association between FGM and other stress-related variables with neuroendocrinological alterations by means of the HCC. To take all possible predictors into account, we approached this question in an explorative manner: First, we used a non-parametric machine learning technique (random forest regression embedded in a conditional inference framework [27]), allowing a large number of predictors to identify the most important predictors. Second, we computed a linear regression model predicting the HCC by identified variables, gaining information on the significance and the directions of associations as well as the general model fit.

## Methods

### Participants

In cooperation with the non-governmental organizations Norwegian Church Aid (NCA), EGLDAM and the Women’s Affairs headquarters in Jijiga, we recruited a convenience sample of 167 women living in various parts of the town. Two (1%) women did not know which FGM group they belonged to, and were therefore excluded in the analysis (*N* = 165). The local partners approached women from different districts in Jijiga and made appointments with those who had agreed to take part in an interview on their experience with FGM and their current mental wellbeing.

Table 1 shows the basic sociodemographic characteristics of the sample. On average, the participants were 30.7 years old (*SD* = 12.6, *range* 13–80) and the majority belonged to the Somali (59%, *n* = 98) or Amharic (30%, *n* = 50; other: 10%, *n* = 17) ethnic groups. In accord with the EDHS 2005 [28], a high percentage of women had endured FGM. During data assessment, we had difficulties finding uncircumcised women—the noFGM group in this study ultimately constitutes a small group (*n* = 18) of women with both manifold vulnerability and resilience characteristics. Therefore, the group cannot be interpreted as pseudo-experimental control group in the final analysis. Most of the participants reported FGM III (48%, *n* = 79) or FGM I (36%, *n* = 60). Only a minority reported FGM II (5%, *n* = 8) or being uncircumcised (11%, *n* = 18). Due to the small number of women reporting FGM II, the latter and FGM III were merged in the analysis. The age and type of FGM depended on the women’s cultural background. Somali women were commonly circumcised according to FGM II/III at the age of 7.6 (*SD* = 2.6, 95% *CI* [7.1; 8.2]) years, with a secular trend towards circumcising girls at earlier ages: Somali women with FGM III aged 26 years and older reported an average circumcision age of 8.2 years (*SD* = 2.7, 95% *CI* [7.4; 9.0]), while the younger group (≤ 25 years) reported an average age of 6.9 years (*SD* = 2.3, 95% *CI* [6.1; 7.7]; *t*(79) = − 2.24, *p* < .05). Somali women who underwent FGM I are a minority (*n* = 14, 14%), and have a younger average age (average age: *M* = 24.5, *SD* = 13.9, 95% *CI* [16.5; 32.5]). Amharic women typically endured the procedure shortly after birth (*n* = 31, 86%); only 5 women reported having undergone FGM later (one each at 3, 5, and 15 years of age, and two at 7 years of age). No particular pattern in terms of circumcision age was found for the FGM cases of the group of Oromo and other ethnicities (*N* = 14).

**Table 1:** Basic information on sociodemographic characteristics, lifetime exposure to childhood familial violence, lifetime traumatic events and mental health outcome measures due to the FGM groups

	No FGM		FGM I		FGM II/III		
	*n* = 18		*n* = 60		*n* = 87		Statistics
Sociodemographic characteristics							
Age *(M ± SD [CI])*	24.8 ± 4.4	[22.6, 27.0]	30.6 ± 12.8	[27.3, 33.8]	32.1 ± 13.3	[29.2, 35.0]^1^	*F*_(2,160)_ = 2.59
Years of education *(M ± SD [CI])*	10.3 ± 4.8	[8.0, 12.7]	8.1 ± 4.3	[7.0, 9.2]^2^	4.1 ± 5.1	[3.0, 5.2]	*F*_(2,161)_ = 19.55***
Ethnicity (%, *n*)							*Chi*^*2*^_(4)_ = 102.69***
Somali	5.6	(1)	23.3	(14)	95.4	(83)	
Amhara	77.8	(14)	58.3	(35)	1.1	(1)	
Other	16.7	(3)	18.3	(11)	3.4	(3)	
Marital status (%, *n*)							*Chi*^*2*^_(6)_ = 10.12
Single	50.0	(9)	28.3	(17)	37.9	(33)	
Married	33.3	(6)	41.7	(25)	21.8	(19)	
Divorced	5.6	(1)	18.3	(11)	21.8	(19)	
Widowed	11.1	(2)	11.7	(7)	18.4	(16)	
CFV (*M* ± *SD* [*CI*])	5.4 ± 3.3	[3.7, 7.0]	6.2 ± 4.1	[5.1, 7.2]	5.6 ± 4.8	[4.5, 6.6]^3^	*F*_(2,161)_ = 0.34
LTE (*M* ± *SD* [*CI*])	0.8 ± 1.2	[0.2, 1.43]	1.4 ± 1.5	[1.0, 1.8]	1.9 ± 1.9	[1.4, 2.3]	*F*_(2,162)_ = 3.28*
Mental health measure							
HCC (pg/mol; *M* ± *SD* [*CI*]))	28.3 ± 19.7	[17.8, 38.8]^4^	29.4 ± 18.3	[23.5, 35.2]^5^	31.6 ± 13.2	[27.8, 35.4]^6^	*F*_(2,103)_ = 0.34
PTSD score (*M* ± *SD* [*CI*])	1.2 ± 4.7	[−1.2, 3.5]	0.8 ± 2.6	[0.2, 1.5]	5.7 ± 10.6	[3.4, 7.9]	*Welch*_(2,43.94)_ = 8.25***
PTSD diagnosis (%, *n*)	5.6	(1)	0	(0)	18.4	(16)	*Chi*^*2*^_(2)_ = 13.49***
ShuD Dissociation score (*M* ± *SD* [*CI*])	0.1 ± 0.3	[− 0.5, 0.3]	1.0 ± 3.1	[0.2, 1.8]	2.3 ± 5.3	[1.2, 3.4]	*Welch*_(2,98.07)_ = 9.16***
Depression score (*M* ± *SD* [*CI*])	18.5 ± 6.7	[15.2, 21.8]	18.2 ± 6.0	[16.6, 19.7]^2^	22.5 ± 11.0	[20.2, 24.9]^1^	*Welch*_(2,51.20)_ = 4.78*
Major Depression (%, *n*)	0	(0)	0 (0) ^2^		12.6	(11)	*Chi*^*2*^_(2)_ = 10.44**
Anxiety score (*M* ± *SD* [*CI*])	11.7 ± 2.5	[10.51, 12.94]	11.6 ± 2.6	[11.0, 12.3]	14.1 ± 6.4	[12.7, 15.5]^3^	*Welch*_(2,56.31)_ = 5.30**
Substance dependence (%, *n*)	11.1	(2)	6.7	(4)	4.6	(4)	*Chi*^*2*^_(2)_ = 1.17
Substance abuse (%, *n*)	0	(0)	1.7	(1)^2^	1.1	(1)	*Chi*^*2*^_(2)_ = 0.34
Suicidal ideation (%, *n*)	0	(0)	8.3	(5)	10.3	(9)	*Chi*^*2*^_(2)_ = 2.06
Psychotic disorder (%, *n*)	0	(0)	0	(0)	2.3	(2)	*Chi*^*2*^_(2)_ = 1.8

*CFV* childhood familial violence, *LTE* lifetime traumatic events

*p ≤ .05, **p ≤ .01, ***p ≤ .001; ^1^*n* = 85, ^2^*n* = 59, ^3^*n* = 86, ^4^*n* = 16, ^5^*n* = 40, ^6^*n* = 50

#### FGM procedural information

Table 2 provides a summary of information on FGM, presenting data on women without FGM and women who underwent FGM I, or FGM II/III, separately. The majority of women reported that they did not agree to their circumcision (*n* = 101, 69%) and that they were not informed prior to FGM as to what would happen (*n* = 133, 91%). During the procedure, 72 (65%) women were blindfolded, and 36 (33%) were gagged. Only a minor portion of women reported having received analgesics (*n* = 36, 25%), which were, however not necessarily effective. The procedure was usually performed using a razor blade (*n* = 86, 74%), or in a few cases, a knife (*n* = 24, 21%). One (1%) woman reported having been circumcised with broken glass and 4 (3%) with scissors. Almost all women (*n* = 96, 92%) who remembered the event reported having experienced intense fear and/or helplessness during the procedure; this did not differ according to the FGM type (*n*_*FGM I*_ = 19, 86.4%, *n*_*FGM II/III*_ = 77, 93.9%; *Chi*^*2*^_*(1)*_ = 1.39, *p* > .05). In the aftermath of FGM II/III, 100% (*n* = 83) of the women reported having had their legs bound for 2 to 40 days (*M* = 9.8, *SD* = 6.8, 95% *CI* [8.3; 11.3]), 56 (*n* = 64%) were isolated for several days (*M* = 11.4, *SD* = 8.1, 95% *CI* [9.2; 13.6]) and 83% (*n* = 72) were deprived of food or drink for up to 30 days (*M* = 9.6, *SD* = 6.4, 95% *CI* [8.1; 11.1]). This was less extensive for women who endured FGM I: 10 (17%) women reported having had their legs bound between 1 to 10 days (*M* = 5.6, *SD* = 2.8, 95% *CI* [3.6; 7.5]), 8 (13%) were isolated up to 20 days (*M* = 11.0, *SD* = 4.8, 95% *CI* [7.0; 15.1]) and 9 (15%) were deprived of food or drink for up to 12 days (*M* = 6.9, *SD* = 3.1, 95% *CI* [4.5; 9.2]).

**Table 2:** Self-reported age at circumcision, location, immediate complications and lifetime physical complaints due to the FGM groups

	FGM I		FGM II/III		
	*n* = 60		*n* = 87		Statistics
Age at circ. (*M* ± *SD* [*CI*])	3.1 ± 4.3	[2.0, 4.2]	7.6 ± 2.7	[7.0, 8.1]	*t*_(89.53)_ = −7.10***
Immediate complications (< 14 days after FGM; %, *n*)					
Severe pain	50%	(11)^1^	84%	(70)^2^	*Chi*^*2*^_(1)_ = 11.63***
Excessive bleeding	5%	(1)^1^	23%	(19)^3^	*Chi*^*2*^_(1)_ = 3.72
Infections/fever	0%	(0)^1^	36%	(30)^2^	
Lifetime chronic physical complaints due to FGM (%, *n*)					
Pelvic pain	10%	(6)	60%	(52)	*Chi*^*2*^_(1)_ = 36.82***
Urination problems	10%	(6)	37%	(32)	*Chi*^*2*^_(1)_ = 13.29***
Menstruation problem	11%	(7)	67%	(58)	*Chi*^*2*^_(1)_ = 43.55***

*p ≤ .05, **p ≤ .01, ***p ≤ .001; ^1^*n* = 22, ^2^*n* = 83, ^3^*n* = 84

### The cycle of infibulation, defibulation, and re-infibulation

Menstruation, sexual intercourse and giving birth typically mean further stages of suffering in the aftermath of FGM—particularly following FGM II/III. In this subsample, 82 (94%) reported having been infibulated. This occurred by means of thorns (*n* = 57, 70%) or thread (*n* = 24, 30%). One woman did not know how she had been infibulated. During menstruation, 51 (62%) women reported having experienced severe pain, 52 (63%) had complications passing blood, and 32 (39%) had fever or infections. A total of 52 (60%) women had had sexual intercourse and were therefore able to answer questions on defibulation: 42 (82%) women were defibulated by a traditional circumciser or another person. For 9 (18%) women, the husband performed defibulation on the night of the wedding. After childbirth, a total of 21 (41%) women reported having been re-infibulated up to 10 times. On average, the vaginal opening had been closed up to 3.4 times (*SD* = 2.7, 95% *CI* [2.2; 4.6]) after childbirth.

### Procedure

All interviews were conducted in a private office at the Women’s Affairs headquarters in Jijiga in July, August and September 2010. Participants were asked to sign their informed consent prior to the interview; none of them refused. For minors (one was 13 years old, six were 16 years old and four were 17 years old), caregivers were invited to sit in during the informed consent; none of them advised their daughters not to take part in the interview and agreed to leave the session following the provision of informed consent. Each participant received 50 Birr ($2.50) as financial compensation. The interview lasted around two to three hours. The study was approved by Vivo International and Norwegian Church Aid (NCA). These are both international non-governmental organizations with long-standing experience in Ethiopia as well as in the Somali Region.

A team of four clinical psychologists (post-doctoral and doctoral, as well as two trained and supervised psychology master-level students) conducted the interviews with the help of English and Amhara and/or Somali-speaking interpreters in all interviews. The latter were trained in a two-day workshop introducing the applied concepts and were advised to translate literally in order to minimize the potential bias or confusion involved with personal interpretation.

### Measures

Sociodemographic information was obtained from each participant by administering a semi-structured questionnaire asking for age, ethnicity, educational background in years and domestic status as well as the participant’s physical health over the previous month (cough, cold/flu, shivering, fever, malaria, headache, pain, diarrhea, vomiting, scabies/skin rash, positive HIV/AIDS test, other physical pain).

To assess details on the individual’s experience with FGM, we constructed a 45-item checklist (*yes/no*). In the first part, general questions regarding, for example, age at circumcision, previous knowledge regarding the tradition and location were asked. Then, with the help of anatomical sketches of the female genitalia, we asked details about the subject’s own FGM, her emotional experiences during the procedure, as well as subsequent complications (short- and long-term). Finally, we asked questions regarding current attitudes on FGM. All items were evaluated separately.

Further traumatic experiences were assessed using the Checklist of Childhood Familial Violence (CFV) [29] and the event checklist part of the Posttraumatic Stress Diagnostic Scale (PDS) [30]. The 36-item CFV assesses childhood traumatic experiences at a physical, verbal-emotional, sexual and witnessed level. We added two items to assess intimate partner violence (*“Have you been beaten by your husband?”* and *“Have you been beaten by the family of your husband?”*). The PDS event checklist contains 12 potentially traumatic event types (e.g., torture, sexual abuse, accident). For both checklists, the sum of all items approved comprised the final scores.

Participant diagnostic status and PTSD symptom severity were assessed using the PTSD Symptom Scale - Interview (PSS-I) [31]. The PSS-I assesses the 17 DSM-IV [32] symptom criteria for PTSD. We assessed the symptom occurrence during the previous month (instead of over the past 2 weeks as specified by the questionnaire manual) to check the diagnostic status and to match the time period of cortisol analysis. Each item was rated on a four-point scale ranging from 0 (*not at all/only once*) to 3 (*five or more times per week/almost always*). PTSD severity was calculated by summing all of the symptom scores (possible scores range from 0 to 51). Internal consistency was excellent in this study (Cronbach’s *α* = .95).

To assess the syndrome of trauma-related dissociation, we applied the Shutdown Dissociation Scale (ShuD) [33]. This 13-item questionnaire assesses sensory deafferentation (e.g., transitory deafness or blindness), reduced nociception/analgesia, numbness, transitory paralysis, loss of speech/suppressed vocalization, pseudo-neurological symptoms, (pre-)syncopes and out-of-body experiences on a four-point scale (0 – *not at all* to 3 – *several times a week*). All items were added to a total score ranging from 0 to 39. Good psychometric properties were also found in the study by Schalinski et al. ([34]; Cronbach’s *α* = .75). In this study, Cronbach’s *α* was .84.

The Hopkins Symptom Checklist-25 (HSCL-25) [35] was used to determine the severity of depression (15 items) and anxiety (10 items). Referring to the last 2 weeks, the subject rated each item on a four-point scale from 1 (*not at all*) to 4 (*extremely*). A separate sum score was computed for the two syndromes ranging from 15 to 60 for depression, and 10 to 40 for anxiety. The HSCL has shown good psychometric properties in previous studies [36–39]. In this study, Cronbach’s *α* was .92 for the depression scale and .86 for the anxiety scale.

Furthermore, we applied the Mini-International Neuropsychiatric Interview (M.I.N.I., 5.0.0, January 1st, 2002) [40] to assess the diagnostic status of major depression (*A. major* depression episode), suicidal ideation (B. suicidality), drug abuse (K. non-alcohol psychoactive substance use disorder) and psychotic disorders (L. psychotic disorder). The M.I.N.I. only takes a short amount of time to administer, and has shown satisfactory concordance with the Structured Clinical Interview for DSM-IV-TR Axis I Disorders – Patient Edition (SCID-P), the Composite International Diagnostic Interview (CIDI) and with expert diagnosis [40].

### Cortisol sampling

In accordance with the laboratory protocol of Stalder et al. [41] and Kirschbaum et al. [16], hair samples were cut from the posterior vertex. We used 1 cm of the segments closest to the scalp for cortisol extraction. Considering the hair growth rate of Africans (0.74 cm/month) [42], the hair segment length of 1 cm roughly signifies 1 month of growth – the time span assessed by the PSS-I. For cortisol determination, 5–10 mg of whole, nonpulverised hair was incubated in 1800 ml methanol for 18 h at 45 °C. Following extraction, cortisol levels were determined using a commercially available immunoassay with chemiluminescence detection (CLIA, IBL-Hamburg, Germany). The intra- and inter-assay coefficients of variation of this assay are below 8% [16]. The mean cortisol level was *M* = 30.2 (*SD* = 16.2, *range* 8.5–92.8), and thus comparable to other African samples [19]. Outlying values (≥ 3 *SD* above mean) were found for two women. In the presented analysis, we excluded the outliers and log-transformed HCC values (positive skewness). A total of 60 (35.9%) women refused to give hair samples because of religious or traditional objections; excluding four cases with missing data in CFV (*n* = 1), FGM (*n* = 1) and age (*n* = 2), the neuroendocrinological analyses include a total of 103 women (sociodemographic characteristics did not differ significantly between these subsamples).

### Data analysis

All missing values were excluded from the analysis (see Table 1). Furthermore, we excluded women who reported having not remembered specific details during their circumcision in the descriptive analysis of the procedure; this was the case for women who reported having been circumcised at an age below 3 years as well as for one woman with FGM I and one and two women with FGM II/III (depending on the item). The descriptive data are presented as frequencies (%), means (*M*) and standard deviations (*SD*). Group differences were analyzed using independent sample *t*-tests and univariate analysis of variance (ANOVA) for continuous variables; post-hoc group differences are indicated by the reported confidence intervals in the tables. Heteroscedasticity was found in certain variables (PTSD, shutdown dissociation, depression, anxiety). Because an *F*-test performed on data with heteroscedasticity would tend to be less conservative, we computed the *Welch* statistic instead. *Chi*^*2*^ tests were used for categorical variables. *Fisher’s Exact* test was applied in cases of cell frequencies less than five. All statistical tests are two-tailed. Pearson’s correlation coefficient was computed as measures of associations for two continuous variables.

As the group of FGM II/III presented with a higher trauma load, we conducted a stepwise linear regression model to investigate the impact of FGM on the association of lifetime traumatic events and PTSD severity. In the first step, we entered FGM to predict PTSD symptom severity; here, FGM was dummy coded with FGM I as baseline (due to low sample size and heterogeneous characteristics, the noFGM cannot be interpreted as a baseline control group). In the second step, the number of lifetime traumatic events types was entered in addition to FGM, and in the third step, the interaction term of the two predictors was entered. To address potential problems caused by heteroscedasticity, we used bootstrapping (second step); all confidence intervals of the models’ standardized estimates were very close to the bootstrapped confidence intervals, suggesting that the model could be regarded as robust to heteroscedasticity. Cohen’s *f*^2^ effect size was computed, with *f*^2^ < .05 constituting a small, *f*^2^ > .15 a moderate, and *f*^2^ > .35 a large effect [43].

For statistical analysis of the hair cortisol data, we first used random forest regression [44] with conditional inference trees (hereafter “conditional inference random forests” or RF-CI) [45] to identify the best predictors of the HCC. Here, we included all variables that would theoretically induce stress (lifetime traumatic events, childhood domestic violence, husband domestic violence, FGM, age at circumcision, PTSD, anxiety, depression, shutdown dissociation, time since the last traumatic event, physical health, HIV/AIDS, age, education). Second, to assess the predictive significance and the direction of associations of these predictors in a parametric model, we conducted a simple linear regression with the most important predictors. The number of predictors (*k*) was set according to the rule of thumb recommended in Tabachnick and Fidel [46] to test a full regression model (50 + 8 *k*) and to interpret individual predictors (104-*k*).

RF-CI is a non-parametric machine learning technique. Unlike the classical random forest [44], the RF-CI does not display a bias towards predictors with many categories in the variable selection process [47]. Following the principles of ensemble methods, a certain number of trees (ntree) are aggregated to compose the random forest. Each tree is built using binary splits of the previously subsampled data (subsampling rate = 63.2%) [27, 48]. The splitting variable is chosen according to the strength of the association between the covariates and the outcome [27, 45] from a set of randomly preselected predictors (*p*, mtry, *p*/3) [49]. Next, the importance of each predictor variable is ranked based on the ensemble of trees (conditional variable importance, *cvi*) [47]. To visualize the results, we built single trees from the whole data set. These are, however, less robust (e.g., biased by outliers) and should not be interpreted without considering the results of the whole ensemble. The PDS event checklist includes a dichotomous item for familial violence and FGM. These events were excluded in the PDS sum score (lifetime traumatic events) applied in the RF-CI computation, as the FGM checklist and the CFV assess these variables more precisely. All cases with missing values in one of the predictor variables were excluded. The analysis was conducted using SPSS 21 and R (version 3.4.1). The implementation we used to compute the RF-CI was cforest [50] from the R package party [51] with unbiased variable selection [45, 50].

## Results

Table 1 illustrates lifetime exposure to violence and mental health problems divided for women without FGM and women who endured FGM I, or FGM II/III, respectively. No group differences were found for childhood familial violence (*p* > .05), but for the number of lifetime traumatic events: women who endured FGM II/III also presented with a higher trauma exposure (*p* < .05).

Accordingly, in the group of FGM II/III, more women fulfilled the criteria of PTSD (FGM II/III: *n* = 16, 18%; FGM I: *n* = 0; noFGM: *n* = 1, 6%). While 11 (12%) women in the FGM II/III group fulfilled the diagnostic criteria of major depression, none of the other groups presented the required severity of symptoms. Furthermore, group comparisons revealed that PTSD and trauma-related symptoms such as shutdown dissociation, depression and anxiety were elevated in women who underwent FGM II/III (group comparisons were significant at 95% levels). Only a few women (< 5%) were diagnosed with substance dependence and/or abuse and/or psychotic disorders; for these, no group differences were found.

### FGM, trauma exposure, and posttraumatic stress

**Fig. 1:**
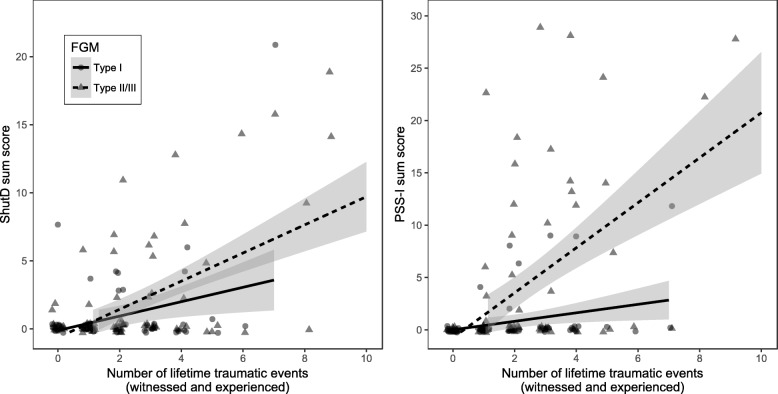
The least square fitted conditional mean (smoothed with 95% CI) of the PSS-I and ShuD total score interacting with the number of lifetime traumatic events according to FGM I versus II/III. *Note:* FGM = female genital mutilation, PSS-I = Posttraumatic Stress Scale – Interview, ShuD = Shutdown Dissociation Scale, More severe forms of FGM are connected with higher levels of PTSD and, to a lesser extent, shutdown dissociation phenomena

Investigating the contribution of FGM to the association of the number of traumatic events and PTSD severity, we conducted a stepwise linear regression model; the results are presented in Table 3. In the first step, FGM significantly predicted PTSD severity, with elevated symptoms in the FGM II/III group (*p* < .001). In the second step, both FGM (*p* < .01) and the number of lifetime traumatic events (*p* < .001) predicted PTSD severity, and the model comparisons indicated a significant improvement in step two (*F*_(1,161)_ = 75.3, *p* < .001). In the third step, in addition to the predictors FGM and the number of lifetime traumatic events the interaction term of the two variables was inserted; the latter became the only significant predictor. The analysis explained 43.7% of variance and model comparisons indicated a better fit than the former (*F*_(2,159)_ = 11.6, *p* < .001). The last step demonstrates that, with having endured FGM II/III, the negative effects of non-FGM-related traumatic stressors are increased, and these women are more susceptible to developing PTSD. Concordantly, Fig. 1 illustrates the interaction of FGM with other lifetime traumatic events and PTSD severity as well as shutdown dissociation.

**Table 3:** Stepwise linear regression analysis predicting the PTSD symptom severity by FGM (dummy coded with FGM I at baseline; step 1), FGM and the number of lifetime traumatic events (step 2) and the latter variables and their interaction term

	*beta* (SE)	*β*	*t*
Step 1 (*F*_(2,162)_ = 7.3, p < .001, adjusted R2 = .07, *f*^2^ = .09)			
(Intercept)	0.83 (1.34)	.09	0.42
FGM: type II/III vs. I	4.83 (1.34)	.54	3.60***
FGM: noFGM vs. type I	0.33 (2.15)	.03	0.15
Step 2 (*F*_(3,161)_ = 32.2, *p* < .001, adjusted *R*^2^ = .36, *f*^2^ = .60)			
(Intercept)	−2.78 (0.96)	−.32	−3.00**
FGM: type II/III vs. I	3.58 (1.12)	.40	3.19**
FGM: noFGM vs. type I	1.81 (1.79)	.20	1.01
LTE	2.68 (0.31)	.69	8.68***
Step 3 (*F*_(5,159)_ = 26.5, *p* < .001, adjusted *R*^2^ = .44, *f*^2^ = .83)			
(Intercept)	0.18 (1.12)	.02	0.16
FGM: type II/III vs. I	−1.21 (1.45)	−.13	−0.83
FGM: noFGM vs. type I	−0.50 (2.12)	−.05	−0.23
LTE	0.47 (0.56)	.12	0.85
Interaction: FGM type II/III vs. I and LTE	3.15 (0.66)	.81	4.76***
Interaction: FGM: noFGM vs. type I and LTE	1.30 (1.38)	.34	0.95

Unstandardized beta coefficients (*beta*) with standard error (*SE*), uncorrected standardized regression coefficients (*β*), and *t*-values (*t*) with *p ≤ .05, **p ≤ .01 and ***p ≤ .001 are reported

*FGM* female genital mutilation, *LTE* lifetime traumatic events

### Cortisol

To identify the most important predictors of the cortisol level, we used RF-CI. Then, we tested the variables in a simple linear regression model.

#### Predictor identification

The *cvi* was ranked as follows: years of education (*cvi* = 26), childhood familial violence (*cvi* = 4), lifetime traumatic events (*cvi* = 3), and FGM (*cvi* = 1) and the age at circumcision of < 1 year (*cvi* = 1). The *cvis* were below 0.5 for the other variables (symptom severity of PTSD, anxiety, major depression diagnosis, shutdown dissociation, and drug abuse, khat consumption, psychosis, the time since the traumatic event, physical health, intimate partner violence, and specific FGM ages beside < 1 years of age).

#### Linear regression

**Fig. 2:**
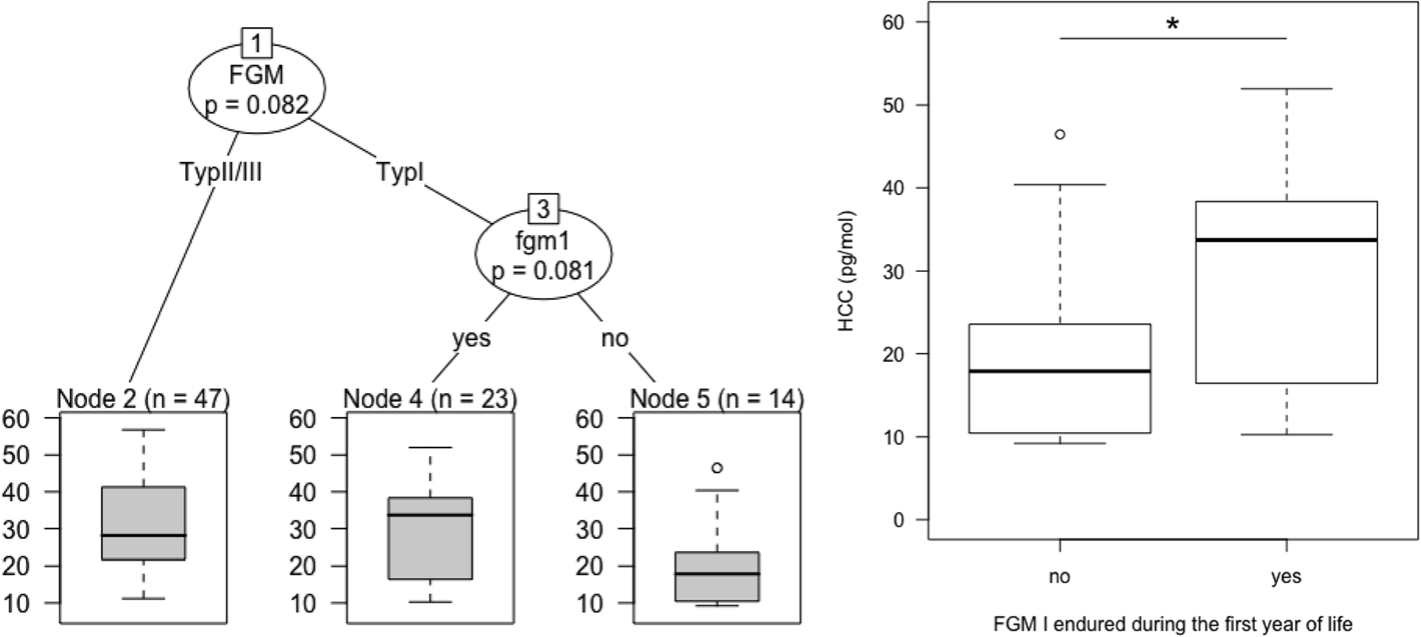
Displays a single tree partitioning the sample according to FGM types and whether the circumcision was endured within the first year of life (only FGM I). Women who endured more invasive forms of FGM, but also women who endured the procedure within their first year of life, present with higher HCC. HCC comparison within the group FGM I revealed significantly higher levels for women who were circumcised in their first year of life (t_(29.18)_ = − 2.18, *p* = 0.038)

On the basis of the variable ranks (*cvi*), the linear regression model included the years of education, family violence during childhood, lifetime traumatic events, FGM (dummy coded with FGM I as a baseline) and whether FGM was endured during the first year of age (yes/no). The model explained 17% of variance with a large effect (*F*_(6,96)_ = 4.5, *p* < 0.001, *f*^2^ = .28). More years of education was associated with lower HCC (*β* = −.08, *p* < 0.01). Regarding the FGM variables, the more invasive forms of FGM was linked with higher HCC (*β* = .12, *p* < 0.01). Interestingly, women who reported having been circumcision during the first year of life also presented with higher cortisol levels (*β* = 0.16, *p* < 0.001; see Fig. 2). Lifetime exposure to traumatic events as well as childhood familial violence did not reach significance. The results are summarized in Table 4. Figure 1 presents a single tree partitioning the sample of women who endured FGM according to FGM types and whether the circumcision was endured within the first year of life (only FGM I, none of the women in the group FGM II/III reported to be circumcised during the first year of life).

**Table 4:** Linear regression analysis predicting hair cortisol levels by education, lifetime traumatic events, childhood familial violence, FGM (dummy coded with FGM I at baseline) and FGM endured during the first year of age

	HCC (mg/mol)		
	*beta* (SE)	*β*	*t*
(Intercept)	2.97 (0.15)	.89	19.26***
Education	−0.03 (0.01)	−.08	−2.73**
LTE	−0.02 (0.03)	−.01	−0.59
CFV	0.02 (0.01)	.04	1.94
FGM: type II/III vs. I	0.41 (0.14)	.12	2.90**
FGM: no FGM vs. type I	0.32 (0.17)	.10	1.86
FGM < 1 year of age	0.52 (0.15)	.16	3.43***

Unstandardized beta coefficients (*beta*) with standard error (*SE*), uncorrected standardized regression coefficients (*β*), and *t*-values (*t*) with *p ≤ .05, **p ≤ .01 and ***p ≤ .001 are displayed. *F*_(6,96)_ = 4.51, *p* < 0.001, *f*^2^ = .28, multiple *R*^*2*^ = .22, adjusted *R*^*2*^ = .17

*HCC* hair cortisol concentration, *CFV* childhood family violence, *LTE* lifetime traumatic events

## Discussion

In this article, we aimed to investigate FGM and its psychopathological sequelae as well as neuroendocrinological associations measured by means of the HCC in an urban sample of women living in Jijiga, the capital of the Ethiopian Somali region. Group differences showed that PTSD and trauma-related symptoms such as shutdown dissociation, depression and anxiety were more severe in the subgroup of FGM II/III. The stepwise linear regression indicated an acceleration of PTSD symptom development with the accumulation of other traumatic experiences, especially for individuals with more severe forms of FGM. Using RF-CI, we found alterations in the HCC according to FGM-related variables as well as to the years of education and childhood familial violence. More invasive forms of FGM as well as enduring FGM within the first year of life was linked to higher levels of cortisol.

### FGM

In line with former studies, almost all women (92%, FGM age ≥ 3 years) reported having experienced intense fear and/or helplessness during their circumcision. Investigating this separately for the FGM groups revealed that, irrespective of the actual FGM type, the procedure applies not only in its objective characteristics to the A-criteria of PTSD as defined in the DSM-5 (severe injury, threat to life, sexual violence), but also involves intense subjective reactions of fear and helplessness.

Regarding the psychopathological sequelae of FGM, we identified women who endured FGM II/III as being particularly vulnerable to developing mental health problems. The results demonstrated that women who underwent FGM II/III suffered more often from (sub)clinical symptoms of PTSD and also reported more trauma-related complications such as shutdown dissociation, depression and anxiety than women who endured FGM I. The stepwise regression analysis revealed an interaction effect of FGM II/III and the number of lifetime traumatic events, indicating that more severe forms of FGM increase the vulnerability to PTSD, such that additional traumatic stressors exert more devastating effects [8]. This aligns with previous findings [5, 10, 11]. Notably, there is a substantial number of women who seem to be resilient (see Fig. 1a); they have a high trauma load, but do not report trauma-related symptoms. Potential resilience factors, such as an increase in social integrity, remain to be explored.

### FGM and hair cortisol

In addition to the psychological variables, we measured long-term neuroendocrinological alterations by means of the HCC to explore potential adaptions of HPA axis activity in conjunction with FGM and other stress-related variables. Therefore, we statistically defined the most influential predictors (using RF-CI) and computed a parametric linear regression model, determining the direction of the associations and providing a significance test for each predictor. The results indicate long-term changes of cortisol levels in association with FGM. As implicated by the HCC as biological marker for stress, women who endured more severe forms of FGM presented with higher levels of hair cortisol. We interpret this result as evidence for the upregulation of the HPA axis activity as a consequence to the traumatic nature of the procedure itself in combination with the continuous cycle of infibulation, defibulation and re-infibulation and other physical consequences. These factors may render women who endured FGM II/III more vulnerable to the adverse effect of traumatic events at the psychological side (see Fig. 1). Furthermore, women who endured the procedure during their first year of age (in our study exclusively FGM type I) showed more pronounced levels of cortisol. This result is in line with previous research on FGM and early life stress. For example, Taddio et al. [52] found an enhanced stress reaction (facial expression, length of time screaming) at vaccination for babies that had been circumcised four to 6 months prior. Scholars agree that neonates, as well as babies in the preverbal phase, are able to respond with the full range of emotional, cognitive and physiological peritraumatic reactions (e.g. palpitation, muscle tension, tachycardia/bradycardia, vasoconstriction/vasodilatation, numbing) [53], and although they are unable to verbalize their experience of the event, the neuronal paths are generated and will be consolidated in the case of further traumatization [54]. The particular relevance of the prenatal period and the early years in life have repeatedly been demonstrated to play a critical role in the (epigenetic) programming of the HPA axis [15, 55] and other negative health outcomes [56].

This study demonstrates that even 20 years after the declaration of FGM as a human rights violation by the UN (A/RES/48/104, Article 2(b)), the practice remains widespread—even in urban regions. The findings show that the awareness of effects of FGM has to go beyond direct somatic complaints and encompass psychopathological risks as well. The provision of adequate health care which particularly addresses the consequences of traumatization is key to overcoming the manifold effects of FGM. Here, evidence-based, culturally sensitive psychotherapeutic tools have been developed to succumb these forms of traumatization, such as Narrative Exposure Therapy [57, 58]. Trauma-specific interventions should complement medical and more holistic approaches, as suggested by Mulongo et al. [59]. Cultural aspects have to be considered for all forms and regions of intervention [60].

### Hair cortisol and other variables

Notably, we did not find a positive relation between other stress-related variables and HCC after controlling for the level of education and FGM-related variables. For instance, Schalinski et al. [21] found higher HCC in a sample of 42 traumatized refugees as well as Simmons et al. [61] who recently published a study with 70 children (mean age 9.5 years). In contrast, Hinkelman et al. [62] found lower HCC in individuals with a history of childhood familial violence in a sample of depressed patients and healthy participants (*N* = 84). Also Kalmakis et al. [63] reported a negative relation in healthy college students (*N* = 55), and Steudte-Schmiedgen et al. [24] in military personnel. Additionally, we did not find an association with age [64] or the number traumatic events [19–21] or major life events [22], respectively. Steudte-Schmiedgen et al. [51] found a negative correlation of HCC with the number of traumatic events and frequency of traumatization. Moreover, contradictory results were previously reported for PTSD (e.g. [19, 23, 65]), which was one of the negligible predictors (*cvi* < 0.5) in the current analyses. Similarly, inconsistent previous results were found for depression [62, 66], which was again irrelevant in the regression analysis of our study. In order to explain associations between HPA axis regulation, cortisol secretion, HCC, and clinical constructs, more research is needed. A better understanding of traumatization during specific windows of opportunity and its effect on the regulation of stress may allow to generate algorithms which eventually could validate subjective psychological reports with biological measures such as HCC.

### Limitations

The lack of a comparable uncircumcised and/or non-traumatized control group constitutes a major limitation in the analysis. Moreover, in regard to the group of FGM I, where the procedure is often not remembered due to the young age at circumcision (≤ 3 years), women had to be excluded from the analysis. Furthermore, the study is correlational in nature, and thus, factors associated with the type of FGM and factors beyond the ritual itself may additionally be responsible for the observed mental health conditions. Also, the reports are retrospective and may therefore be biased, as most of the cross-sectional psychological studies. Despite the anatomical sketches, the FGM type was identified based on self-reported information and may thus not be reliable for all women; medical examinations were not feasible within the present study. Finally, the sample is not representative and generalizations may not necessarily hold up; in particular, we assume that there is a selective exclusion of women with high avoidance symptoms and other concerns regarding FGM, mental health, or research in general. To our knowledge, this is the first study associating FGM with neuroendocrinological outcomes, therefore a replication study especially including HCC would be of particular interest.

## Conclusion

All forms of FGM may have negative health outcomes. Women who endured extensive forms of FGM present with a higher risk of developing mental disorders. They report more severe symptoms of PTSD and trauma-related problems, including shutdown dissociation, depression and anxiety. These findings are corroborated by heightened levels of cortisol in this group. Furthermore, women who endured FGM I during their first year of life showed higher concentration of cortisol, despite the lack of more obvious mental health implications. Evidence-based trauma interventions have been developed (e.g. [57]), and in the next step must be scaled up along with integral treatment approaches to mitigate the negative consequences of FGM.

## Abbreviations

CFV: Childhood familial violence
FGM: Female genital mutilation
HCC: Hair cortisol concentrations
HSCL: Hopkins Symptom Checklist
LTE: Lifetime traumatic events
M.I.N.I.: Mini-International Neuropsychiatric Interview
PSS-I: Posttraumatic Stress Symptom Scale – Interview
PTSD: Posttraumatic stress disorder
ShuD: Shutdown Dissociation Scale
